# A Call for Epidemiological Research on Myeloid-Derived Suppressor Cells in Ovarian Cancer: A Review of the Existing Immunological Evidence and Suggestions for Moving Forward

**DOI:** 10.3389/fimmu.2019.01608

**Published:** 2019-07-09

**Authors:** Ashley E. Stenzel, Scott I. Abrams, Kirsten B. Moysich

**Affiliations:** ^1^Department of Cancer Prevention and Control, Roswell Park Comprehensive Cancer Center, Buffalo, NY, United States; ^2^Department of Immunology, Roswell Park Comprehensive Cancer Center, Buffalo, NY, United States

**Keywords:** myeloid-derive suppressor cells (MDSCs), epidemiology, STAT (signal transducer and activator of transcription), IRF8 transcriptional coactivator, ovarian cancer

## Abstract

Recently, there have been encouraging findings suggesting that myeloid-derived suppressor cells (MDSCs) may be a good target for studying immune suppression in ovarian cancer. MDSCs are an abundance of immature myeloid cells that have demonstrated the ability to decrease tumor-infiltrating immune cells, increase the accrual of tumor-associated macrophages and regulatory T cells, as well as secrete various pro-inflammatory mediators and growth stimulating cytokines. Most studies on this topic utilized murine models, but there are limited reports in human subjects which have important limitations. With the majority of ovarian cancer patients presenting with distant metastases and a corresponding 5-year relative survival rate of < 30%, continued efforts are obligatory toward identifying potential prognostic factors. Given the difficulty of studying exposures in this patient population, as well as the existing immunologic characteristics of this cancer, there is growing interest in further identifying genetic and immunologic associations with patient survival. Furthermore, prognostic factors that may necessitate therapeutic intervention may significantly alter disease outlook. In this review paper, we address the current literature on MDSCs and their immunosuppressive behavior in ovarian cancer patients. While the previous studies on these cells in ovarian cancer have demonstrated some potential prognostic significance, there are many limitations to such studies including small sample sizes, inconsistent staging and histology, as well as inconsistent surface markers for the identification of MDSCs. Additionally, such studies include minimal patient characteristics involved with the clinical course of ovarian cancer. Here, we have proposed improving on studies analyzing MDSCs as a potential prognostic factor in ovarian cancer patients, as well as further identifying the potential of this novel prognostic factor in future care, through the use of a comprehensive epidemiologic model.

## Introduction

Ovarian cancer is a rare, yet fatal disease. This cancer is found to be more prevalent in Caucasian women, with a median age at diagnosis of 63 (1). While it is not one of the higher incident cancers, it is the 5th leading cause of cancer-related deaths among women. Incidence has slightly decreased over time, however, mortality remains high. The 5-year survival rate for this malignancy is 46.5%, overall, but when broken down by stage, those with distant metastases have a 5-year survival rate of <30%. This is important to acknowledge as the majority of women with this disease (60%) have progressive disease with distant metastasis at initial presentation. This is largely due to the vague characteristics of symptoms for this disease, including, but not limited to: bloating, dyspepsia, early satiety, changes in urinary habits, and generalized pelvic pain and discomfort (2, 3). Such symptoms are frequently disregarded, as they can be explained by many non-malignant etiologies. Due to this, many women will not present for evaluation by their clinician until persistence of such symptoms, or may not experience such symptoms until late stage disease. Additionally, many women with ovarian cancer not only have widespread disease at diagnosis, but also present malignant ascites which is an indicator of poor prognosis.

The most common diagnosis for this disease is epithelial ovarian cancer (EOC), which can be further broken down by histotype to high grade serous, endometrioid, clear cell, mucinous, and low grade serous EOC. Of note, while many women will have a good initial response to tumor debulking and chemotherapy treatments, many women will have disease recurrence, develop treatment-resistant disease, and eventually succumb to their disease. For these reasons, it remains of high importance to continue exploring potential risk factors, as well as prognostic factors, for EOC.

At present, there are a handful of well-established risk factors for this malignancy. Reproductive risk factors such as early age at menarche, late age at menopause, post-menopausal hormone replacement therapy use, endometriosis, and nulliparous status have all been strongly supported in the literature (4–7). Other established and increasingly supported risk factors include smoking, physical inactivity, and BMI. There is currently a panel of pathogenic mutations with a significant association of risk with developing ovarian cancer, as well as known hereditary cancer syndromes, with the level of increased risk varying across specific mutations and syndromes (2, 8). An area of research with increased interest has been the role of immune suppression in EOC etiology and prognosis.

## Immunosuppression in Cancer

Cells of the immune system are derived from various progenitor cells within the bone marrow that differentiate into a diverse range of mature cell types that ultimately comprise all lineages of the hematopoietic compartment. Such cell populations are programmed to provide effective host defense, which includes those with activating, as well as suppressive or phenotypes (9–11). In normal tissue, such suppressor cells are known to play key roles in regulating the immune response in response to pathogens or tissue repair following injury and damage. However, in several solid tumors as well as hematologic malignancies, there exist populations of suppressor cells that are thought to play major roles in creating a tumor-promoting or submissive microenvironment. Tumor-associated immune cells of myeloid origin (i.e., monocytic or granulocytic), for example, may occur from either an abundance of immature myeloid cells due to dysregulated myelopoiesis or myeloid cells that do not function properly (11). Examples of mature, dysfunctional myeloid populations include tumor-associated macrophages (TAMs), tumor-associated neutrophils (TANs), and tolerogenic dendritic cells (tolDCs). TAMs and TANs have been associated with tumor progression by promoting chronic inflammation in the tumor microenvironment, and have been found in abundance at multiple stages of cancer (11–13). TAMs are a subset of activated macrophages that become tumor-promoting in the tumor microenvironment (TME) via polarization from a functional, anti-tumor M1-like into an M2-like macrophage. Such macrophages have the ability to promote chronic inflammation via inflammatory cytokines, VEGF production for angiogenesis, as well as upregulation of insulin-like growth factor-1 (IGF-1) for invasion, and metastasis. These cells also express cytokines such as IL-10, subsequently aiding in suppression of tumor infiltrating lymphocytes (11). TANs are thought to play a role in secreting various chemokines that draw TAMs to the TME, as well as being associated with increased platelets which may play a role in the TME for maintenance of tumor health. TAMs and TANs have been associated with worse prognosis in EOC, noted to not only be associated with an increase in VEGF expression and tumor vascularization, signaling an increase in matrix metalloproteinases (MMPs), increasing tumor progression through aiding the disruption of the basement membrane and increasing the cellular mobility of ovarian tumor cells for metastasis (11, 12). It is thought that a portion of the aforementioned cell types are derived from immature myeloid-derived suppressor cells (MDSCs), or are stimulated by MDSCs (9, 10). Recently, there has been an increasing amount of evidence for the role of MDSCs in various cancers, including EOC.

MDSCs include both monocytic and granulocytic subtypes (9, 10). They have demonstrated potential to produce multiple chronic-inflammatory and mediators that support tumor growth, invasion, and metastasis. They have also been described as having the potential to suppress antigen-specific T cell responses through multiple mechanisms such as lacking MHC antigen expression, synthesis of chronic-inflammatory cytokines and mediators, including IL-10, arginase-1, transforming growth factor beta (TGF-β), and indoleamine 2,3-dioxygenase (IDO), all of which may play integral roles in tumor progression.

While MDSCs are thought to play a role in cytokine production, research has also suggested the role of cytokines in MDSC recruitment (14–16). For example, interleukin-8 (IL-8) has been implicated as a potential player in the accumulation of MDSCs in the tumor microenvironment. Such research has shown the CXCL/CXCR pathway and, IL-8 production in particular, suppresses immune infiltrating cells, and increases MDSC activity, with high amounts of granulocytic MDSC activity noted in these studies. IL-8 can be produced by cancer cells within the tumor, demonstrating the cancer's ability to evade apoptosis through increasing these immunosuppressive MDSCs (14, 16). Specifically, IL-8 has been linked to neutrophil extracellular trap (NET) formation within granulocytic MDSC populations which may aide in the angiogenesis of a tumor promoting microenvironment (16).

In addition to suppressing T cell-mediated immune responses, these cells have also been associated with expanding the regulatory T cell population, which also act to suppress effector T cells (17–19). These above-mentioned mechanisms could explain the ability of MDSCs to suppress both the adaptive and innate immune responses in cancer, among other diseases. Lastly, MDSCs are noted to have increased expression of the programmed death ligand 1 (PD-L1), known to downregulate T cell function through engagement of cell surface PD-1 (17). Additionally; studies among other neoplastic disease have demonstrated an inverse relationship between MDSCs and tumor-infiltrating lymphocytes (TILs) (17). This suggests a complex relationship between TILs and MDSCs. These immunosuppressive pathways of MDSCs have led to considerable interest in measuring circulating MDSC levels as a potential prognostic factor in cancer. That is to say those individuals who have a higher accumulation of MDSCs are thought to have increased risk of progression of their malignancy, and worse overall survival. Additionally, targeting MDSCs in EOC may be a potential area for immunotherapeutic approaches in the future.

## MDSC Regulation in Neoplastic Disease

In an effort to understand MDSC accumulation in cancer, a number of studies have analyzed potential genetic and molecular factors. Several studies have reported interferon regulatory factor-8 (IRF-8), as well as the STAT family of transcription factors (STAT1, STAT3, STAT5, STAT6), as having potential roles in their development (11, 20–24). IRF-8 has been shown to be downregulated, resulting in increased levels of MDSCs (20–24). This is due to its presumed role in regulating the myeloid differentiation during hematopoiesis. Ordinarily, this particular transcription factor positively regulates progenitor differentiation to functional monocytes, macrophages, and dendritic cells, indicating that a loss of or a reduction in the expression of IRF8 may result in impaired myeloid differentiation and the production of aberrant, or immature myeloid cells with MDSC characteristics (20). IRF-8 can be induced by IFN-γ under pro-inflammatory conditions, which has an established role with activating antitumor immune responses.

Epigenetic silencing of IRF-8 in human tissue, as well as mouse models, was shown to increase the accumulation of MDSCs (25, 26). Lee et al. conducted a study assessing methylation of promotor CpG islands, resulting in the silencing of IRF-8 in human tissues of multiple carcinomas (25). Their results demonstrated that silencing IRF-8 led to the loss of IFN-γ stimulation, a known immune response-inducing cytokine. Waight et al. demonstrated that tumor-induced downregulation of IRF-8 led to an accumulation of MDSCs (26). Additionally, these investigators also noted a reduction of MDSCs when they utilized mouse models with IRF8 overexpression, indicating that not only does a loss of function of this transcription factor lead to the accumulation of such suppressive cells, but overexpression of IRF-8 as an interventional application may necessitate further research for potential clinical implication in reducing the amount of accumulated MDSCs. Lastly, these researchers addressed the role of the STAT family of transcription factors in increasing the accumulation of MDSCs by analyzing STAT3 and STAT5 activity in this process. Their results demonstrated that activation of STAT3 or STAT5 can downregulate IRF-8 expression, providing a molecular explanation for why such STATs influence the accrual of these suppressor cells. Multiple studies have demonstrated the association of the STAT family of transcription factors with the increased accumulation of MDSCs across many different malignancies and various disease models (27–30). Essentially, the aforementioned studies conclude that when the STAT3/5 pathway is upregulated and the expression of IRF-8 is downregulated, an increased accumulation of MDSCs is anticipated, demonstrating the strong role of these transcription factors in regulating the MDSC accumulation, and proper development of myeloid cells.

## MDSC Expression and Murine Ovarian Cancer

In murine models of ovarian cancer, results have demonstrated multiple potential factors influencing the expansion of MDSCs. In a study by Zhao et al. investigators evaluated depletion of SORBS2, a protein coding gene for sorbin and SH3, and its impact on the tumor microenvironment (31). When they utilized a knockdown murine model for SORBS2, they observed increased metastatic behaviors of the ovarian tumors, and noted increased MDSC levels and M2 (suppressive) polarization of TAMs. Subsequently, they reported decreased survival among the mice with SORBS2 knockdown, thus concluding that SORBS2 plays a role in suppressing the invasion of ovarian tumors. Interestingly, they did note the possibility for reversing such metastatic characteristics by forced expression of growth inhibitor protein coding gene WFDC1, and/or IL-17D, a gene that codes for cytokine production/stimulation, both of which bound to SORBS2 to decrease metastatic potential in this study.

Similarly, the EMT transcription factor, Snail, was evaluated in a knockdown model (32). By knocking down this transcription factor an increase in tumor infiltrating immune cells and a decrease in MDSCs were observed. These researchers speculated that this may be due in part to the relationship between Snail and the CXCL/CXCR pathway. This pathway is associated with cytokines that play a role in recruitment of MDSCs to the tumor site, and may be upregulated by Snail. Therefore, these investigators concluded that the promotional recruitment of MDSCs via the CXCL/CXCR pathway may be inhibited by Snail knockdown.

Previous studies have demonstrated that diminishing MDSC populations in ovarian tumor ascites was associated with decreasing the levels of IL-10 (33). Further studies on this topic using mouse models have demonstrated the ability of IL-10 production by MDSCs, suggesting that IL-10 may be a product of these cells via changes in CD62L and lymphocyte acting gene, LAG-3. They also report that these processes aide in creating positive feedback by IL-10 stimulating further MDSC expansion and immune suppression.

**Figure 1:**
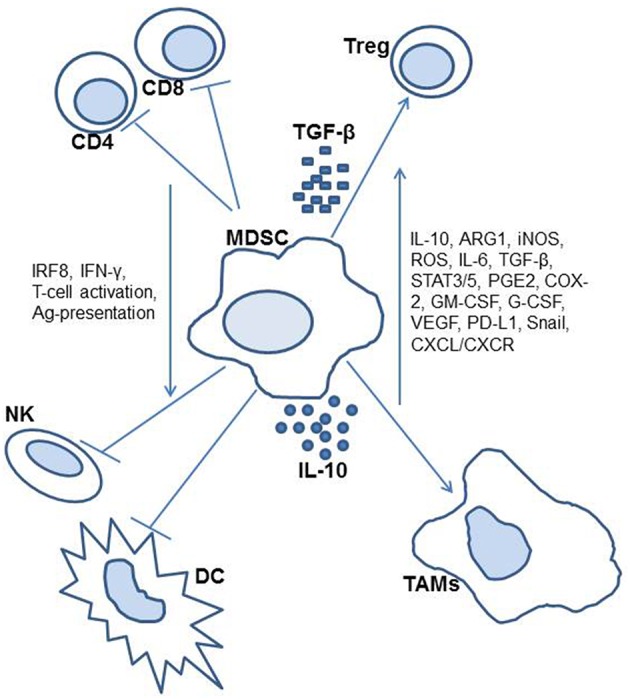
Diagram of myeloid-derived suppressor cell activity in the cancer patient.

Lastly, murine ovarian cancer models have led to the discovery that MDSCs may accumulate and develop in environments without NADPH oxidase, a component that was previously thought to be a factor in such cellular processes (34). This was previously thought to be due to the association of reactive oxygen and nitrogen species in environments with accumulating MDSCs. However, this was not the case in a study of NADPH defective mice, which still demonstrated the ability to accumulate suppression from the MDSCs, therefore was found to be independent of NADPH oxidase. Other murine studies on this topic have been conducted more closely related to therapeutic research on these suppressor cells, and as such are included in the later section on potential therapies. Figure 1 includes an illustration of identified MDSC activity.

## MDSC Expression and Human Ovarian Cancer

As previously stated, various factors affecting MDSC expansion have been studied in multiple types of cancers and across various biological models. Like many other carcinomas, high levels of MDSCs in human EOC have also been associated with poor prognosis (13, 35–41). In human EOC models, various factors influencing the etiology of MDSC expansion in this disease are still under active investigation. Horikawa et al. studied the relationship between high levels of VEGF expression and accumulation of MDSCs in high grade serous ovarian cancer patients, as well as mouse models (36). Their results demonstrated a statistically significant increase of immune suppression (characterized as downregulation of lymphocytes) in patients with high levels of VEGF expression. Additionally, they reported significantly worse overall and progression free survival, among those with high VEGF compared to those categorized as having low VEGF expression. They then proceeded to test the correlation between VEGF expression and MDSC levels among their mouse models, finding that high expression of VEGF was significantly associated with MDSC expression, and inversely associated with lymphocytic expression. Additionally, the MDSCs were shown to have increased VEGFR2 expression. They also reported that VEGF–A appeared to be directly correlated to MDSC differentiation and migration, with VEGFR/VEGF-dependent recruitment to the tumor site.

As stated above, recent studies in EOC have demonstrated the potential association between MDSCs and the upregulation of IGF1, which may promote both proliferative activity among the cancer cells, as well as migration for invasion and metastasis among these cells (13). Other studies have focused on ascites fluid and MDSCs, such as levels of interleukin-6 and-10 (IL-6, IL-10) in the ascites, suggesting that IL-6 and IL-10 in the ascites fluid may contribute to the expansion or function of MDSCs in EOC patients (37). Likewise, IL-1β has also been reported to have an association with increased levels of MDSCs in EOC patients when compared to healthy controls (39). It has been noted that in EOC, specifically, there appears to be inhibition of MDSCs recruitment to a tumor microenvironment lacking chemokine receptor CCR2. Additionally, TAM migration was explored in a population of patients with samples extracted from tumor or ascites fluid (40). The TAMs were then analyzed for CCR2 mRNA expression. Their results suggested that TAMs with defective CCR2 expression also demonstrated inhibited migration to the tumor, however TAMs were able to overcome this in the presence of complement component 5 (C5a).

If TAMs are in fact upregulated by and/or differentiating from MDSCs, then this would explain the potential role of CCR2 in both MDSC and TAM inhibition. Additionally, a tumor-associated inflammatory mediator, prostaglandin E2 (PGE2), has demonstrated a role in controlling the expression and interactions of CXCL12 and its respective receptor, CXCR4, which are implicated in the process of tumor progression (42). These interactions showed increased expression of CXCR4 on monocytic MDSCs, with PGE2 inducing CXCL12 in the tumor microenvironment, as well as CXCR4 on MDSC precursor cells. PGE2 was seen to induce COX2 expression, which further stimulates PGE2, thus having developed a positive feedback loop to continue the accumulation of MDSCs. Another study also identified that accrual of MDSCs may be associated with increased DNMT3A (involved in DNA methylation) in a PGE2 positive cellular environment (43). This suggests that downregulating DNMT3A may improve prognosis with regard to MDSC activity. Lastly, a study by Santegoets et al. analyzed a monocytic MDSC to dendritic cell ratio to evaluate its usefulness as a prognostic factor among EOC patients after treatment (44). They reported this ratio as being an independent potential prognostic factor for EOC survival, with high levels of monocytic MDSCs being correlated with higher risk of mortality.

While the aforementioned studies have confirmed that MDSCs as a prognostic factor in EOC patients represent an area worth studying, the methodologies employed across studies demonstrate significant differences. As shown in Table 1, the research studies performed on human samples to date had several limitations including; small sample sizes, unspecified/diverse histotypes of EOC, variable staging, inconsistent source of collection (i.e., blood, tumor, ascites), and inconsistent use of surface markers for the identification of MDSCs. Many of the studies do not report on MDSC subset analysis within this cancer. Furthermore, to our knowledge, there are no studies published from an epidemiological/population science perspective. Implementing a study design with a well-defined patient population, clinical characteristics, consistent MDSC surface markers, and a larger sample size would allow us to draw more definitive conclusions on the value of MDSCs as a prognostic factor in this patient population. It would also be of great value to compare blood measurements to ascites fluid levels of MDSC activity in a larger sample size.

**Table 1:** Summary of previous human studies on MDSC activity in EOC patients.

**Author**	**Sample size**	**Histotype**	**Stage**	**MDSC collection**	**Controls**	**Surface markers**
Horikawa et al. (36)	56	HGSOC	III, IV	Ascites fluid, pre-chemotherapy/ radiation	None	CD33+, CD11b+
Wu et al. (37)	31	Serous, mucinous, endometrioid, mixed	I–IV	Peripheral blood, sera, ascites, pre-chemotherapy/ radiation	31 age-matched, healthy peripheral blood donors	CD14+HLA-DR–/low
Huang et al. (40)	Not specified	Multiple solid tumor cancers, unspecified ovarian	Not specified	“Tumor and blood,” “untreated”	None	Lin– HLA-DR–
Obermajer et al. (42)	24	Unspecified epithelial ovarian cancer	III, IV	Ascites and sera, prior to any adjuvant therapy	None	CD11b+
Rodriguez-Ubreva et al. (43)	22	Unspecified epithelial ovarian cancer	III, IV	Ascites and blood, untreated	10 healthy donors' blood	CD11b+CD33+ CD34+
Santegoets et al. (44)	36	Unspecified epithelial ovarian cancer	Not specified	After treatment with tocilizumab, carboplatin/doxorubicin or gemcitabine and interferon-α 2b	“Healthy donor blood”	CD14-CD15- double-negative (dn) CD33+CD11b+ and CD33-CD11b+

Additionally, as there is a growing interest in understanding the functionality of MDSCs and their mechanisms in EOC progression, there have also been efforts put toward understanding potential genetic variation and MDSC activity. In a large consortium study, single nucleotide polymorphisms (SNPs) in 24 genes with presumed relationships to MDSC expansion were analyzed for their association with survival among ovarian cancer patients, which showed no significant associations for SNP variations (45). However, it is worth noting that many smaller studies, such as those previously mentioned (13, 36, 40, 42), have demonstrated the potential of genetic expression and interactions in the accumulation of MDSCs in the tumor microenvironment, therefore, further epidemiological research focused on gene-environment interactions may be warranted.

## Potential Future Directions

**Figure 2:**
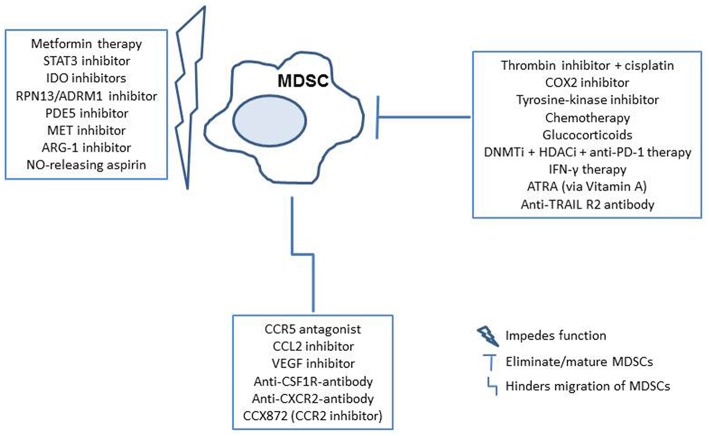
Potential therapeutic implications for MDSC targeted therapy in the cancer patients. MDSCs can be targeted by elimination/maturation, impeding function, or hindering migration. Therapies are outlined in blue, with subsequent immune reactions in shapes that follow (35, 41, 46–55).

In addition to studying the underlying characteristics of MDSCs in EOC, recent efforts have demonstrated the potential application of anti-PD-1 after anti-Gr-1 MDSC depletion therapy, as well as other immunotherapies that may effectively reduce MDSCs or interfere with their activity in ovarian cancer models to allow activation of a tumor-infiltrating immune response (35, 41). Interestingly, one group of researchers explored Metformin, a pharmaceutical commonly prescribed for diabetic patients, in EOC patients with high MDSC levels (46). They observed decreased levels in both granulocytic, as well as monocytic subsets of MDSCs, which was believed to have occurred due to restriction of adenosine generation. Other studies utilizing murine models have identified glucocorticoids, various checkpoint blockades, direct thrombin inhibitors, DNA methyltransferase (DNMTi)/histone deacytlase inhibitors (HDACi), and RPN13/ADRM1 inhibitors, all demonstrating their potential to alter the levels of MDSCs, or function (47–51). Such approaches have the general goal of enhancing overall survival, though are not yet demonstrated in human subjects. In addition to studies in ovarian cancer models, research on potential therapies to eliminate, impede function of, or inhibit MDSC migration to the tumor microenvironment include; PDE5 inhibitors, STAT3 inhibitors, tyrosine-kinase inhibitors, chemotherapies, CCL2 inhibitors, CCR5 antagonists, VEGF inhibitors, IDO inhibitors, COX2 inhibitors, MET inhibitors, ARG-1 inhibitors, NO-releasing aspirin, ATRA, CCR2 inhibitors, anti-CXCR2 antibodies, anti-TNF-related apoptosis-inducing ligand (TRAIL) death receptor R2 antibody, and with use of an anti-CSF1R antibody. (52–55) Furthermore, specific anti-MDSC antibodies that target surface MDSC proteins are of interest (53). All of these studies suggest that further clinical analysis of such drug applications and reduction of MDSCs in patients with EOC may improve outcomes, and are worth exploring. A brief overview of the mechanisms of MDSC interference by these potential therapies is demonstrated in Figure 2.

There is increasing interest in the role of MDSCs in the etiology and prognosis of cancers. While there is growing evidence building an association between MDSCs and EOC, there is still a wide range of unknown mechanisms and interactions necessitating further research on this topic. Given the above information, one can understand the intrigue in therapeutic intervention with regard to MDSCs in EOC. Developing an extensive understanding on this topic may allow further development of clinical interventions targeting such cellular involvement. However, the limited number of studies on these cells in human EOC has significant caveats. While a recent review paper on MDSCs in gynecologic malignancies was published, this paper was more so highlighting technical aspects of this topic and was not necessarily specific to ovarian carcinoma (56). While their review paper provides a thorough overview of the role of MDSCs as a whole, including examples from multiple malignancies, they do not provide detailed information on the various interactions of MDSC activity in ovarian cancer patients specifically. These authors focus on the overall evolution of MDSC data, and offer insight on potential targeting of MDSCs in general. Due to the minimal existing prognostic and therapeutic factors in this patient population, we feel it deserves special attention. Inclusion of characteristics such as histotypes sub-analysis, analysis of subsets of MDSCs, clinical and epidemiological patient characteristics, and a larger sample size would give rise to more conclusive data. An analysis of this topic utilizing a comprehensive epidemiological model would benefit the field of epidemiology, as well as clinical gynecologic oncology, to fully understand the value of collecting MDSC measurements for patient outcomes, and potential modifiable factors to reduce accumulation of MDSCs in EOC patients.

## Author Contributions

This review article was written by KM and AS, with substantial input from SA.

### Conflict of Interest Statement

The authors declare that the research was conducted in the absence of any commercial or financial relationships that could be construed as a potential conflict of interest.
